# Impact of predator model presentation paradigms on titi monkey alarm sequences

**DOI:** 10.1007/s00265-022-03250-1

**Published:** 2022-10-03

**Authors:** Mélissa Berthet, Geoffrey Mesbahi, Cristiane Cäsar, Klaus Zuberbühler

**Affiliations:** 1 Institute of Biology, University of Neuchâtel, Neuchâtel, Switzerland; 2 Department of Comparative Language Science, University of Zurich, Affolternstrasse 56, 8050 Zurich, Switzerland; 3 Center for the Interdisciplinary Study of Language Evolution (ISLE), University of Zurich, Zurich, Switzerland; 4 INRAE, FERLUS, 86600 Lusignan, France; 5 Vale S.A., Nova Lima, Brazil; 6 School of Psychology & Neuroscience, University of St Andrews, Scotland, UK

**Keywords:** *Callicebus nigrifrons*, Model presentation, Playback experiment, Semantics, Syntax, Vocal reaction

## Abstract

**Abstract:**

Predator presentation experiments are widely used to investigate animal alarm vocalizations. They usually involve presentations of predator models or playbacks of predator calls, but it remains unclear whether the two paradigms provide similar results, a major limitation when investigating animal syntactic and semantic capacities. Here, we investigate whether visual and acoustic predator cues elicit different vocal reactions in black-fronted titi monkeys (*Callicebus nigrifrons*). We exposed six groups of wild titi monkeys to visual models or playbacks of vocalizations of raptor or felid. We characterized each group’s vocal reactions using sequence parameters known to reliably encode predatory events in this species. We found that titi monkeys’ vocal reactions varied with the predator species but also with the experimental paradigm: while vocal reactions to raptor vocalizations and models were similar, felid vocalizations elicited heterogeneous, different reactions from that given to felid models. We argue that subjects are not familiar with felid vocalizations, because of a lack of learning opportunities due to the silent behaviour of felids. We discuss the implication of these findings for the semantic capacities of titi monkeys. We finally recommend that playbacks of predator vocalizations should not be used in isolation but in combination with visual model presentations, to allow fine-grained analyses of the communication system of prey species.

**Significance statement:**

It is common to present prey species with predator models or predator calls to study their vocal reactions. The two paradigms are often used independently, but it remains unclear whether they provide similar results. Here, we studied the vocal reactions of titi monkeys to calls and models of raptors and felids. We show that titi monkeys seem to recognize the vocalizations of raptors but not those of felids. The study of the vocal reactions emitted when titi monkeys cannot clearly identify the threat allows us to draw accurate hypotheses about the meaning of titi monkeys’ alarm utterances. We argue that playbacks of predator calls should be used in conjunction with model presentations, which can allow us to better investigate the information and the structure of the alarm systems.

**Supplementary Information:**

The online version contains supplementary material available at 10.1007/s00265-022-03250-1.

## Introduction

Anti-predator vocal behaviours are often used as a gateway to investigate nonhuman communication systems. Contrary to calls emitted in more subtle social contexts, the stimulus eliciting alarm calls (i.e. the predator) is easy to identify, the behaviour of the receivers is obvious and dependent on the predator’s features (e.g. species, size, location) and the alarm calls are easy to discriminate from the rest of the vocal repertoire (Macedonia and Evans 1993; Zuberbühler 2009). All these logistic advantages make alarm calls an ideal candidate to begin to investigate semantics (the message), syntax (the structure of the message) and the cognitive mechanisms underlying predator-related communication in nonhuman animals (see, for example, Dezecache and Berthet 2018).

Predator presentation experiments are widely used to systematically investigate alarm utterances. They recreate a predatory situation, which is usually rarely witnessed under observational conditions, while controlling for specific factors (e.g. predator species, location or posture). Predator presentation experiments usually take two forms: presentation of visual stimuli (predator models) and playback of auditory stimuli (predator calls).

Presentations of visual stimuli effectively trigger realistic anti-behavioural responses in mammals (e.g. Blumstein 2000; Cäsar 2011), because many mammalian species rely heavily on visual detection of predators (Coss et al. 2005). For example, field experiments show that several species react strongly to leopard-like spotted coat patterns (Coss et al. 2005; Schel and Zuberbühler 2009; Mehon and Stephan 2021) or are able to quickly detect camouflaged or concealed snakes (Shibasaki and Kawai 2009; Isbell and Etting 2017). However, model presentations are difficult to conduct, especially on wild animals, because they are subject to many constraints. First, it must be ensured that the model possesses the visual features that trigger an appropriate anti-predator response (e.g. Mehon and Stephan 2021). Second, it must be presented in a realistic way (but see Ramakrishnan and Coss 2000). For example, the visual stimuli must be placed coherently in the habitat (e.g. on a branch or in the air for raptor models). The model, thus, must be placed in advance in a spot where it can easily be detected by the prey (e.g. on their regular paths, near their favourite feeding spots, etc.), which implies detailed knowledge of the study population. Third, the subjects should not realize that they are being fooled, which implies that they do not get used to the experiment and do not associate the presence of predator models to that of researchers. Trials must remain short and sufficiently spaced to match the rate of natural encounters with the real predator. The observers must often stay out of sight, in particular if working with non-habituated subjects. Finally, other species may detect the visual models and alert the study subjects before the beginning of the experiment (Fallow and Magrath 2010). Due to all these constraints, the rate of failed trials is often high, and model experiments often require intensive months of effort from the researchers.

Since prey mostly react to the vocalizations of predators as if a threatening event was occurring (Hettena et al. 2014), playbacks of predator calls are considered a good alternative to presentations of visual models. Playbacks require the researchers to hide a speaker in a relevant location, out of sight of the subjects, and broadcast good quality recordings of predator vocalizations. As a result, they are easier and usually faster to conduct than model presentation experiments. For that, they are often recommended in field studies (Zuberbühler and Wittig 2011), provided that the predator is not a silent species (e.g. snakes).

Despite their wide use, it is uncertain whether playbacks of predator calls can be considered valid substitutes for presentations of visual stimuli, as it remains unclear whether both paradigms elicit similar vocal reactions in the subjects. Usually, authors compare the reaction of subjects after playbacks of predator vocalizations to a baseline before the experiment or to a control condition (conspecific alarm calls or sounds from the habitat) (Hettena et al. 2014). These methods can confirm that prey react to a predator’s vocalizations, but they prevent us from concluding that prey react as if this specific predator was present. Such a conclusion can only be reached when comparing the vocal reactions of prey after playbacks of predator calls to those after model presentations. This methodology was used in a few studies, which often found that the vocal reactions were slightly different. For example, common marmosets call less when exposed to leopard calls than to leopard models (Kemp and Kaplan 2011). Black-capped chickadees produce different alarm calls to live or models of raptors than to raptor calls (Billings et al. 2015). Sportive lemurs do not emit alarm calls in response to predator call playbacks (Fichtel 2007). Campbell’s monkeys emit fewer and different alarm calls when hearing a predator’s calls than when seeing it (Ouattara et al. 2009a, b, c, d), although the longer exposure to leopard models than to leopard vocalizations may account for this result. Vocal responses of male putty-nosed monkeys are shorter for leopard models than for leopard playbacks (Arnold et al. 2008); while the sequence types given to leopards and eagles are similar between model presentations and call playbacks, their fine structure was not investigated.

Several explanations have been put forward to explain these differences. Billings et al. (2015) postulate that visual stimuli provide direct and reliable information about the predation event, like the predator’s behaviour or location, while acoustic stimuli provide more ambiguous information, which can lead to weaker reactions. Ouattara et al (2009a) suggest that visual stimuli are detected at a shorter distance than acoustic cues: this more imminent threat can increase the arousal level of the callers, which can impact their vocal reactions (see Briefer 2012). They add that acoustic cues can also be heard by the entire group, so individuals may feel less inclined to inform conspecifics with alarm vocalizations. They also postulate that, since most predators are silent when they hunt, the emission of predator calls may suggest that the predator is not hunting or has not detected the prey yet (Ouattara et al. 2009a). Schel et al. (2009) finally suggest that visual direct encounters are a precondition for alarm calling in some species.

These mixed results have also questioned whether prey recognize their predators’ vocalizations. While some species show innate reactions to predator calls, even if extinct in the study area (Li et al. 2011; Hettena et al. 2014; Makin et al. 2019), or can quickly learn the vocalizations of newly introduced predators (Berger et al. 2001; Gil-da-Costa et al. 2003), some species do not seem to recognize their current predators’ calls (Blumstein 2000; Friant et al. 2008; Hettena et al. 2014; Deppe 2020). These differences could be explained, in part, by the vocal behaviour of the predator: with the exception of some predatory birds, most predators do not vocalize while hunting (Barrera et al. 2011), which can represent a lack of opportunity to learn a predator’s call. For example, mule deer do not respond to playbacks of mountain lions, but do respond to playbacks of coyote calls; this could be due to the fact that felids, contrary to canids, are mostly solitary and vocalize on rare, specific occasions (Smallwood 1993; Macarrão et al. 2012; Leuchtenberger et al. 2016).

Another layer of difficulty comes from the possibility that prey react to vocalizations of predators because of their novelty or acoustic properties, rather than because they recognize the predator. This is illustrated by the fact that prey sometimes respond with threat-related behaviours to vocalizations of completely unknown predators (i.e. predators that share no ecological or evolutionary experience with the prey) (Hettena et al. 2014; but see Blumstein 2006).

In sum, the assumption that model presentations and playbacks of predator vocalizations elicit similar vocal reactions is fragile. This can lead to debatable conclusions if playbacks of predator calls are the only way to investigate the alarm vocalizations of a species (e.g. Langmore and Mulder 1992; Zelano et al. 2001; Fichtel and Kappeler 2002; Arnold and Zuberbühler 2006; Stephan and Zuberbühler 2008, 2014; Schel et al. 2009; Greig et al. 2010). Indeed, alarm sequences can encode a large variety of information, from the urgency of the situation to the class, location, behaviour or size of the predator (see review in Dezecache and Berthet 2018). They do so using a variety of different encoding mechanisms: the composition of the vocal reaction, the order or repetition of elements and the temporal variations are some of the many encoding strategies observed in animals (see review in Engesser and Townsend 2019). If crucial information about the predatory situation (e.g. the predator type or its location) is not retrieved by the caller in playbacks experiments, the sequence will be altered. For this reason, it is crucial to assess whether the subjects understand what predator is present, and react accordingly, when exposed to predator vocalizations. If the subjects react differently to predator models and playbacks of predator vocalizations, this can provide an invaluable opportunity to conduct fine-grained analyses on the cognitive mechanisms underlying the production of alarm vocalizations, and their impact on the semantics and syntax of vocal utterances. However, such detailed investigation is often lacking.

Here, we investigate the extent to which the experimental paradigm of predator presentations impacts the vocal reactions of a prey species. Our study focuses on black-fronted titi monkeys (*Callicebus nigrifrons*), which possess a sophisticated alarm system. Specifically, they emit long sequences that are composed in their first parts of two soft calls, the A- and B-calls (Cäsar et al. 2012a), and then gradually switch to a mix of loud call and soft call syllables (Caselli et al. 2014). These alarm vocal reactions are accompanied by mobbing, freezing or fleeing behaviours (Cäsar 2011). Using model presentation experiments, previous studies have shown that several sequence parameters related to the order and composition of the soft-call sequence convey information on the predatory event, such as the predator type (terrestrial vs aerial predator) or location (ground vs canopy), which is understood by listeners (Cäsar et al. 2012a, b, 2013; Berthet et al. 2019b; Narbona Sabaté et al. 2022). Interestingly, these studies also showed that the distance between the predator and the monkeys is not encoded in the sequence (Berthet et al. 2019b; Narbona Sabaté et al. 2022). Finally, social information, such as the identity or composition of the group, is encoded by order and composition parameters of the later soft-call sequence (Narbona Sabaté et al. 2022).

Our study investigates whether alarm sequences elicited by playback of predator vocalizations also encode for predator type and location, using similar encoding mechanisms as those highlighted during model presentations. In most model presentation experiments, one individual spots the stimulus and calls alone, until it is joined by its conspecifics, which makes it possible to isolate single individual contributions. Playbacks of predator vocalizations, on the other hand, are performed simultaneously on all members of the group, making it impossible to isolate individual contributions and preventing a fine analysis of the alarm sequence structure. As such, we focussed our analysis on metrics that do not need to disentangle individual sequences. We also extended our analysis to the whole sequence, i.e. beyond the soft-call sequence.

We compared the vocal reactions of titi monkeys to models and vocalizations of two predator species, a raptor and a felid, presented on the ground and in the canopy. If playbacks of predator vocalizations are equivalent to model presentations, then we expect the titi monkeys to produce a vocal reaction specific to raptors and another specific to felids, regardless of the experimental paradigm.

## Methods

### Study site and subjects

The study was conducted at the RPPN Santuário do Caraça, MG, Brazil (20° 05′ S, 43° 29′ W). This private natural heritage reserve of 110 km^2^ is composed of transition zones between native Atlantic Forest, “cerrado” (savannah), “campo rupestre” (rocky grassland) and “capoeira” (secondary growth vegetation), ranging from 850 to 2072 m in altitude (Brandt and Motta 2002; Talamoni et al. 2014). The central part of the reserve comprises two forests of interest for this study, Tanque Grande and Cascatinha, located 1 km apart from each other at an average elevation of 1300 m (Jarvis et al. 2008). The climate is tropical, characterized by a rainy, hot season (October to March) and a dry, colder season (April to September) (more details in Berthet et al. 2021).

The study population was composed of four titi monkey groups inhabiting the Tanque Grande forest (A, D, R and S groups) and two titi monkey groups inhabiting the Cascatinha forest (M and P groups). Titi monkeys typically live in family groups comprising an adult heterosexual pair, monogamous for life (Dolotovskaya et al. 2020), and up to four offspring. Both sexes disperse after reaching sexual maturity, at around 3 to 4 years of age (Bicca-Marques and Heymann 2013). Group composition is given in Online Resource 1. The A, D, M, P and R groups were habituated to human presence between 2003 and 2008, while the S group was habituated in 2015 (Cäsar 2011; Berthet 2018). At the time of the study, all groups were completely habituated.

### Predators

The Santuário do Caraça is a conservation hotspot for the local fauna. About 70 mammal species (Talamoni et al. 2014) and 300 bird species (Vasconcelos et al. 2003; Cäsar 2011) inhabit the area, including mammalian and avian predators of *C. nigrifrons* (Cäsar 2011; Bicca-Marques and Heymann 2013; Dolotovskaya et al. 2019). In this study, we investigated the vocal reactions of titi monkeys to models and vocalizations of two predator types, a raptor and a felid, present at the Santuário do Caraça.

For the raptor condition, we presented titi monkeys with taxidermy models and vocalizations of a Southern caracara *Caracara plancus*. The caracara is a Falconidae that has an extremely large and diverse habitat, ranging from open to semi-open areas in south Nearctic and Neotropical regions (Ferguson-Lees and Christie 2001). The caracara has a diverse social system, with some individuals being solitary and others living in couples or family parties of less than 5 individuals (Ferguson-Lees and Christie 2001). Very little is known about the vocal behaviour of the caracara, but they emit several different calls, mostly used for social interactions or to signal intruders near the nest (Ferguson-Lees and Christie 2001; Schlee 2007). The caracara has one of the most varied diets among the falconids, as it feeds on carrions and human refuse, and preys birds, insects and small mammals (Travaini et al. 2001; Sazima 2007; Vargas et al. 2007). Even though the rate of predation of titi monkeys by caracaras is unknown, they probably represent a threat to titi monkeys as they can hunt infant howler monkeys (McKinney 2009). Moreover, raptors are the main predators of South American monkeys (Ferrari 2009): encounters with any falconiform, including caracaras, elicit strong anti-predator reactions from titi monkeys (Cäsar 2011; Cäsar et al. 2012a, 2013; Berthet et al. 2019b), probably due to a “better safe than sorry” strategy (Ferrari 2009). Official density reports do not exist, but caracaras are common at the Santuário do Caraça (Vasconcelos and Melo Júnior 2001) and can also be seen or heard several times per day, while flying over or perching in the forest patches of the Santuário do Caraça (MB, pers. obs.).

Due to logistic reasons (namely, the lack of availability of realistic visual models and good quality recordings from the same cat species), we used two species of the Ocelot lineage (*Leopardus* genus) for the felid condition.

We used vocalizations of an ocelot (*Leopardus pardalis*). Ocelots are found in forests of southern Texas, the coasts of Mexico, Central America and the Northern and central regions of South America (Sunquist and Sunquist 2002). They are solitary and active mostly at crepuscule and night. They feed on small mammals, birds and reptiles and sometimes prey on larger mammals like howler monkeys, capuchins, muriquis or titi monkeys (Sunquist and Sunquist 2002; Wang 2002; Abreu et al. 2008; Ferrari 2009; Silva-Pereira et al. 2011; Dolotovskaya et al. 2019). Ocelots are found in the Santuário do Caraça (Talamoni et al. 2014). There is no published data on the density of this species in the reserve, but ocelot population density varies widely from 2.5 to 160/100 km^2^, making it one of the most common felids in South America (Paviolo et al. 2015).

We used a taxidermy model of southern tiger cat (*Leopardus guttulus*). Contrary to ocelots, which have been largely investigated, little is known about the ecology of the southern tiger cats (Wang 2002; Tortato et al. 2021). It was formerly considered a subspecies of oncilla *Leopardus tigrinus*, but was recently reclassified as a distinct species (Trigo et al. 2013). It occurs in Southeast Brazil, mostly in Atlantic forests. It is a solitary cat that feeds on small preys weighing below 100 g like small mammals, birds and lizards (Wang 2002; Silva-Pereira et al. 2011; Oliveira et al. 2016) but can sometimes prey on small primates like marmosets (Ferrari 2009) or mammals weighing > 1000 g (Oliveira et al. 2016). The southern tiger cat is predated by ocelots, which results in the “Ocelot effect” (de Oliveira et al. 2010): where ocelots are present, the southern tiger cat is rare (less than 15 individuals per 100 km^2^, against a density of 13–25/100 km^2^ in areas where ocelots are absent or very rare) (Oliveira et al. 2016) and becomes more active during the day, to avoid ocelots (de Oliveira et al. 2010). Southern tiger cats have never been officially reported in the Santuário do Caraça, but the taxidermy model we used in the study was a roadkill in the reserve in 2008, and one of the authors also spotted a live individual (CC, pers. obs.). It is thus likely that southern tiger cat is present in very low density in the reserve due to the presence of ocelots.

Even if the felid model and vocalizations are not from the same species, we assumed that titi monkeys should react similarly if they recognized predator vocalizations. Indeed, the ocelot and the southern tiger cat are two closely related felid species that both inhabit the Santuário do Caraça and have similar appearance and similar ecology (Sunquist and Sunquist 2002; Silva-Pereira et al. 2011; Castello 2020). Although literature on vocal repertoires of wild cat species is scarce, *Leopardus* species seem to possess similar vocalizations, including meowing, hissing and snorting (Peters 1983; Castello 2020). It is then likely that titi monkeys adopt a “better safe than sorry” strategy when encountering a *Leopardus* species: any stimuli that resemble a dangerous predator (like an ocelot) should elicit anti-predator behaviours, at least in the first seconds of exposure. For all these reasons, it is likely that titi monkeys adopt a similar vocal reaction when exposed to ocelot’s vocalizations and southern tiger cat taxidermy model.

### Model presentation experiments

All the vocal reactions elicited by model presentation experiments were presented in Berthet et al. (2019b). Details of the experimental paradigm can be found in the original publication but are summarized below.

We used three taxidermy predator models as stimuli: one southern tiger cat *Leopardus guttulus* and two caracaras *Caracara plancus*. Each predator species was presented twice to each group: once on the ground and once in the canopy. The order of presentation was randomized across groups. Presentations were separated by at least 10 days for each group. Before each trial, we monitored subjects for at least 30 min and made sure that no duet, group encounter, loud call from a lost individual or predator encounter occurred in the 30 min preceding the experiment.

For the canopy condition, models were realistically placed on branches using a transparent fishing line, at 3–10 m high (mean: 6.2 m). Distance of detection (i.e. distance between the first caller and the model at the time of emission of the first alarm call) varied from 3 to 17 m (mean: 9.2 m). Observers stayed as far as possible from the model during the experiment and did not hide or manipulate it before all monkeys had left the area, to avoid association between the experiment and the researchers.

For each presentation, we recorded the number of individuals involved, i.e. the number of individuals who visually spotted the predator at some point during the trial.

We considered a trial as failed if recording quality was insufficient (cicadas noise; *n* = 1), if model detection took place during setup (*n* = 4), if the model was detected by an individual of less than 2 years old (*n* = 2), if another species gave alarm calls before visual detection by subjects (*n* = 2) or if an individual bumped into the model before detection (*n* = 1). If a trial was scored as failed, we waited for at least 2 months before we retested the group. One experiment (caracara in the canopy, D group) failed three times, and we decided not to rerun the experiment a fourth time. We conducted a total of 23 valid trials between May 2015 and August 2016.

### Playback experiments

For the raptor condition, we used vocalizations of caracara *Caracara plancus*. For the felid condition, we used vocalizations of an ocelot *Leopardus pardalis*.

Ocelot vocalizations were recorded from a captive adult ocelot, Rhaburn, held at the Belize zoo. Vocalizations were recorded by Rhaburn’s zookeeper while the ocelot was feeding (growls). Due to logistical constraints, the feeding growls were recorded using a smartphone’s internal microphone at close distance from the animal, in a MP3 format. A total of 6 min (362 s) of growls and hisses were recorded, divided into 11 sequences. We created 12 ocelot playback stimuli lasting 20–25 s (mean 22.08), each comprising a unique combination of growls and hiss. Each playback stimulus was then normalized at − 1 dB.

Caracara vocalizations were downloaded from Xeno-canto.org (Planqué and Vellinga 2005), a collaborative database hosting recordings of bird vocalizations under Creative Commons licenses. We used five reliable, good-quality MP3 recordings (total duration: 250.52 s), composed of various vocalizations of *Caracara plancus*, and created 12 caracara playback stimuli lasting 20–22 s (mean: 20.83), each comprising a unique combination of vocalizations extracted from the 5 raw recordings. One stimulus also comprised a wing noise (bird flying off). Similar to the ocelot stimuli, each raptor stimulus was normalized at − 1 dB. All recording manipulations (edits, normalization) were conducted using the Audacity software (Audacity Team 2014).

Playback experiments were conducted between January and August 2016. Each group was presented with a unique set of four stimuli corresponding to two predator types occurring in two different locations (raptor in the canopy, raptor on the ground, felid in the canopy, felid on the ground). The presentation of stimuli was randomized among groups. Playback experiments were separated by at least 5 days (mean: 25.10 days) within a group. Each stimulus was only broadcasted once to avoid pseudo-replication.

For each trial, an Anchor AN-Mini loudspeaker (audio output, 30 W; frequency response, 100 Hz to 15 kHz) connected to an iPhone 4.2.1 was covered with a camouflage net and positioned on the ground (“ground” condition) or hung in a tree with a transparent fishing line, at a height of 3–11 m (mean: 8 m) (“canopy” condition). We held the volume of the loudspeaker at a constant level, matching the natural volume of the predators to a human ear. To test the setup, the territorial call of a white-shouldered fire-eye (*Pyriglena leucoptera*) was played once. This bird call is common in the study area and elicits no reaction from the monkeys.

We made sure that no monkey was able to see the speaker nor the preparation of the experiment. We monitored the group at least 30 min before and after the experiment. During the 30 min before a trial, we made sure that no duet, group encounter, loud calls from a lost individual or predator encounter occurred; otherwise, we waited for a further 30 min. We made sure that no vocalization was emitted before the onset of the playback, to avoid interference with the experiment. We made sure that the group was at the same height or below the speaker in the canopy condition, so that the sound was coming from the canopy from their point of view. When all conditions were met, stimuli were played. The distance between the closest individual and the speaker varied from 7 to 20 m (mean: 12.9 m).

For each experiment, we recorded the number of individuals involved, i.e. the number of subjects located within a 6-m radius around the microphone, whose vocal reaction could reliably be recorded.

One trial (caracara ground, A group) failed due to the emission of feeding calls right before the onset of the broadcast. This trial was rerun after a 5-day pause. We conducted a total of 24 valid playback trials.

### Recording equipment

Vocal reactions of subjects were recorded in WAV format with a Marantz solid-state recorder PMD661 (44.1-kHz sampling rate, 16-bit accuracy) and a directional microphone Sennheiser K6/ME66 or K6/ME67 (frequency response, 40 to 20,000 Hz ± 2.5 dB).

### Dataset

We coded the vocal reaction of individuals (*N* = 47 sequences). To this end, we used the vocal repertoire established by Cäsar et al. (2012a). The two main alarm calls emitted in reaction to a predator presence are the A-call and the B-call. C-calls can also rarely occur in the alarm sequence.

We labelled each call emitted within the first 20 s of each experiment. This was defined as the 20 s following the onset of the first predator call or the 20 s following the moment the first individual spotted the predator model. This 20-s duration coincides with the mean emission of 10 calls (18.2 s, Berthet et al. 2019b), which should be considered sufficient to convey reliable, urgent information to conspecifics.

We extracted seven sequence parameters (later referred to as “variables”) from each vocal reaction: (i) the type of the first call emitted, (ii) the proportion of A-calls (the number of A-calls / the number of calls emitted in the first 20 s), (iii) the proportion of B-calls (the number of B-calls / the number of calls emitted in the first 20 s), (iv) the number of calls emitted within these first 20 s and (v) the total duration of the vocal reaction (i.e. the time needed for all monkeys to stop calling at the stimulus or to leave the area). We noted (vi) whether the group emitted loud vocalizations: to this end, we used the titi monkeys’ loud call repertoire established by Caselli et al. (2014) and coded whether A, B or C syllables were emitted during the experimental trial. Finally, we calculated (vii) the proportion of responding individuals (the number of responding individuals among the ones involved / the number of individuals involved).

If no monkey vocally reacted to the experiment (i.e. zero calls emitted), we coded the proportion of responding individuals, the first call, the proportion of A- and B-calls in the first 20 s, the number of calls emitted in the first 20 s and the total duration as 0.

For each sequence, we coded the contextual parameters, including the social parameters (group identity and the number of individuals involved), and the experimental parameters, (predator type [raptor vs felid], location of the predator [ground vs canopy] and experimental paradigm [playback vs model presentation]).

### Statistical analyses

The point of our analysis was to investigate whether the vocal reactions of monkeys could be separated into distinct clusters (i.e. whether we could observe distinct “types” of vocal reaction) and what experimental or social parameters best explained the classification.

We first conducted a dimension reduction to reduce the number of variables to a smaller number of transformed, uncorrelated, important variables that still contain most of the information from the original dataset. This step is crucial to ensure that the final classification is stable and reliable. Since our dataset was composed of both quantitative and qualitative variables, we conducted a factor analysis for mixed data (FAMD) (Husson et al. 2010). Since FAMD cannot be performed on dataset with missing values (NAs), we implemented the six missing values of our dataset with the regularised iterative FAMD algorithm, where missing values are imputed with the mean of the variable (quantitative variables) and the proportion of the category for each category (qualitative variables), calculated from the non-missing data (Audigier et al. 2016).

We then conducted a hierarchical clustering in order to group similar vocal reactions, based on the reduced number of variables (i.e. the results of the FAMD). To this end, we conducted hierarchical clustering on principal components (HCPC). The HCPC we conducted was unsupervised, meaning that the algorithm chose the optimal level for division based on the growth of inertia between clusters (see Husson et al. 2010, 2017).

We investigated which main sequence variables and contextual parameters contributed most to the division into clusters: we used a chi-squared test indicating what variables and parameters were significantly correlated with the clustering (*p* < 0.05).

We finally characterized each cluster using v-tests. A v-test is a standardised deviation between the mean of individuals in a category and the population’s average. Negative v-test values indicate that the population in the category has a lower mean than that of the population, and positive v-test values indicate a higher mean. *p* values can be derived from the v-test using the normal distribution: each cluster is characterized by all variables that are significantly correlated with the vocal reactions composing it (*p* value < 0.05) (more details in Husson et al. 2017). We used v-tests to calculate the degree of correlation between (i) a variable and a cluster, in order to describe each vocal reaction, and (ii) a contextual parameter and a cluster, in order to describe the context in which these vocal reactions are emitted.

Statistical analyses were conducted on R version 4.1.0 (R Core Team 2021). The missing values of the dataset were imputed with the missMDA package (Josse and Husson 2016), and the FAMD and HCPC analyses were performed with the packages FactoMineR (Lê et al. 2008) and factoextra (Alboukadel and Mundt 2020).

## Results

Details of the vocal reactions are presented in Online Resource 2.

**Fig. 1:**
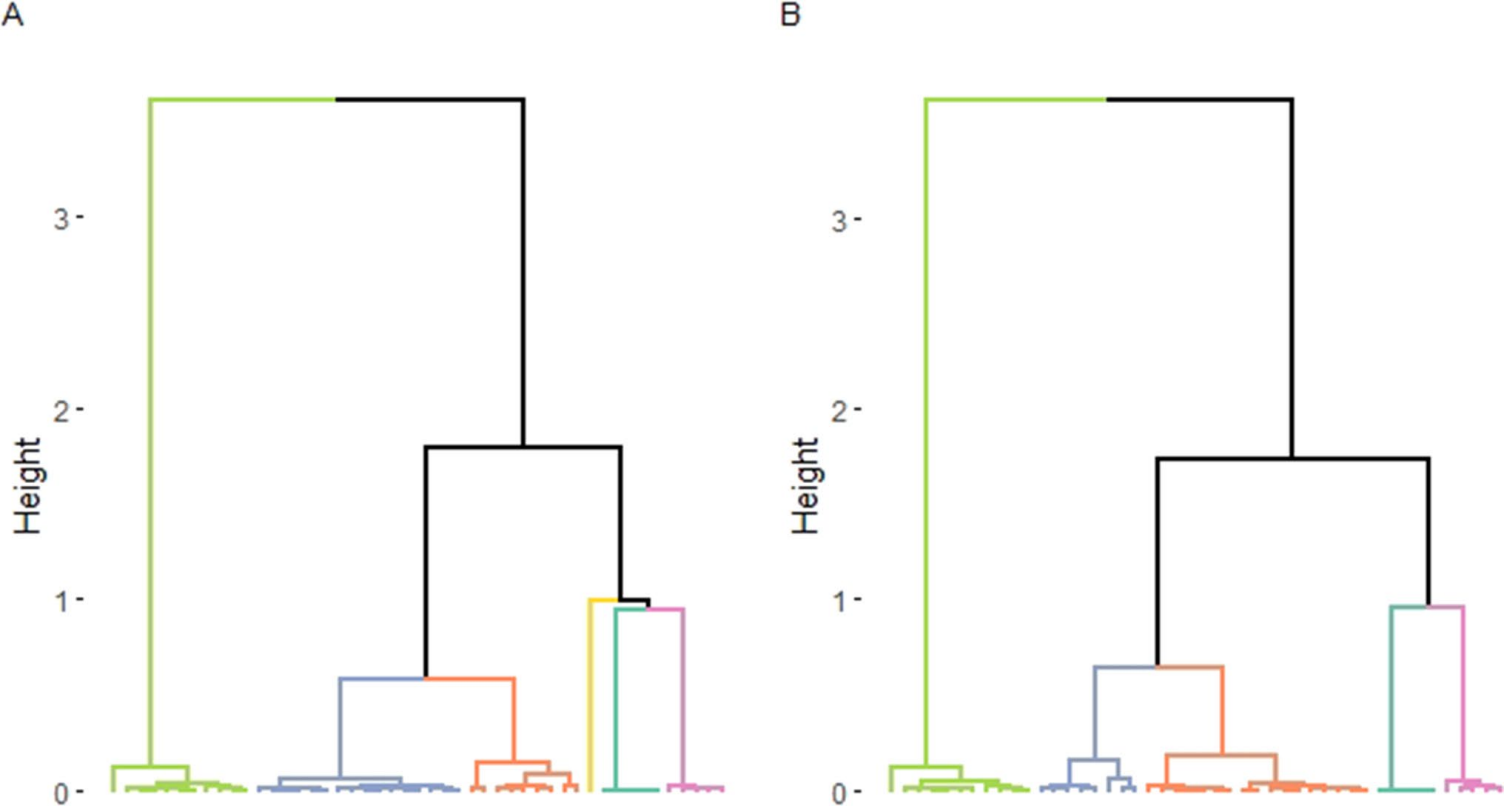
Dendrogram of **A** the unsupervised clustering, which partitioned the data into 6 clusters, and **B** the unsupervised clustering on the updated dataset (i.e. the solitary datapoint was removed), which portioned the data into 5 clusters

A factor analysis for mixed data generated a good representation of the vocal reactions: the five final components expressed more than 90% of the variance in the data (48.89% + 23.21% + 11.17% + 7.48% + 3.57% = 94.32%). Vocal reactions were initially clustered into six groups (Fig. 1A). However, since one of the groups was composed of only one vocal reaction (playback raptor on the ground, P group), we considered this vocal reaction as an outlier and removed it from the dataset.

**Fig. 2:**
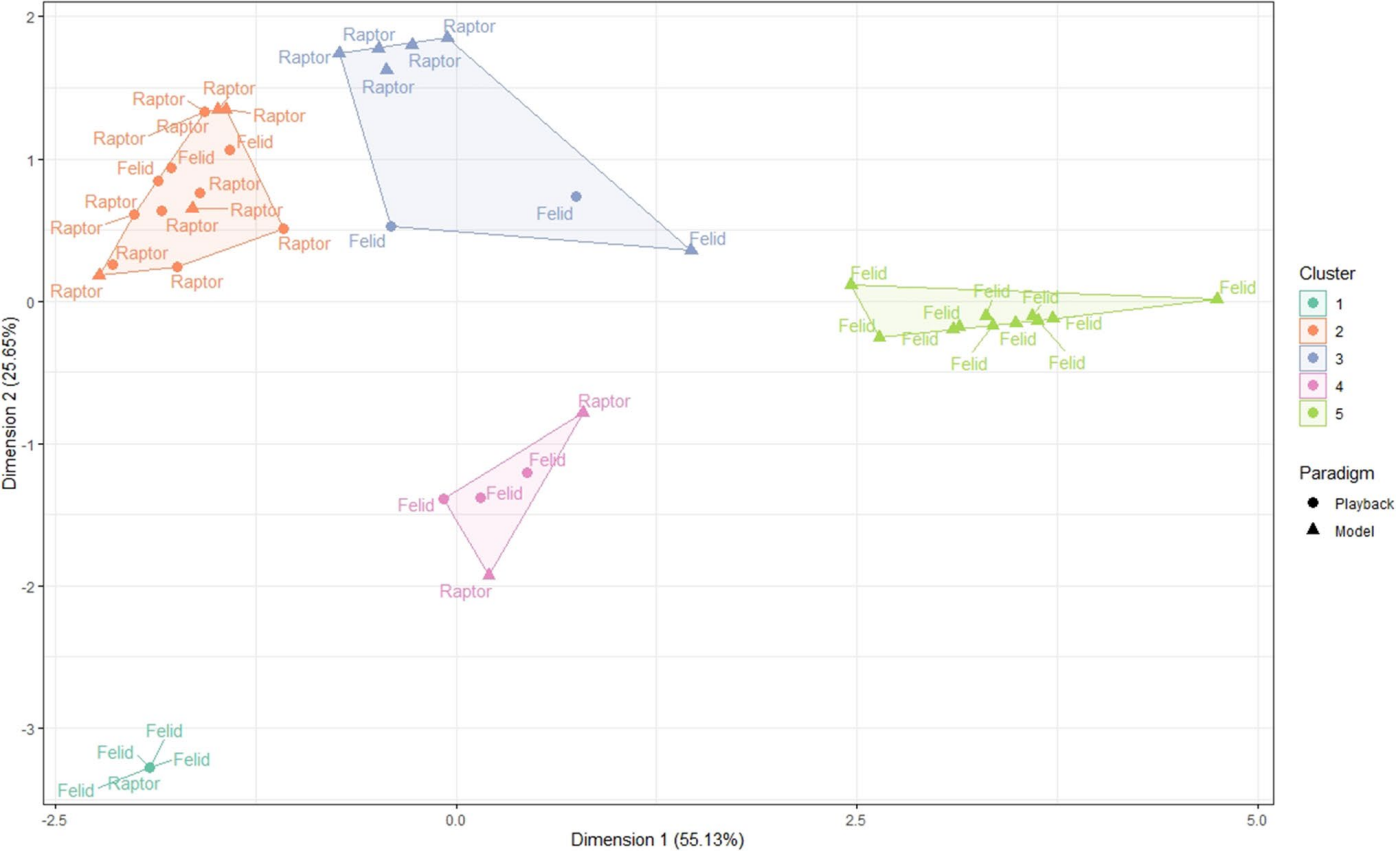
The five clusters of vocal reactions of titi monkeys, depending on the predator type (point’s label) and the experimental paradigm (point’s shape)

As a result, the factor analysis for mixed data conducted on the updated dataset allowed for a better representation of the vocal reactions: the five final components expressed more than 95% of the variance of the data (55.13% + 25.65% + 8.63% + 4.07% + 2.93% = 96.40%). Vocal reactions were clustered into five distinct groups (Figs. 1 and 2, Table 1, Online Resource 3).

**Table 1:** Contextual parameters and sequence variables characterizing each cluster

Clusters		Descriptive variables							Context of emission				
Id	Number of sequences	First call	Proportion of A-calls	Proportion of B-calls	Number of calls	Duration	Loud vocalizations	Proportion of responding individuals	Group identity	Number of individuals involved	Predator type	Predator location	Experimental paradigm
1	5	*No first call *(*v-test* = *4.95*, *p* < *0.01*) **A-call** (**v-test = − 2.43**, ***p* = 0.01**)	**v-test = − 2.40**, ***p* = 0.02**					**v-test = − 4.73**, ***p* < 0.01**					*Playback (v-test* = *2.25*, *p* = *0.02*) **Model presentation **(**v-test = − 2.25**, ***p* = 0.02**)
2	17	*A-call *(*v-test* = *4.98*, *p* < *0.01*) **B-call **(**v-test = − 3.98**, ***p* < 0.01**)	*v-test* = *4.86*, *p* < *0.01*	**v-test = − 4.20**; ***p* < 0.01**	**v-test = − 3.03**, ***p* < 0.01**	**v-test = − 2.82**, ***p* < 0.01**	*No *(*v-test* = *4.57*, *p* < *0.01*) **Yes **(**v-test = − 4.57**, ***p* < 0.01**)			**v-test = − 2.24**, ***p* < 0.02**	*Raptor *(*v-test* = *3.52*, *p* < *0.01*) **Felid **(**v-test = − 3.52**, ***p* < 0.01**)		*Playback *(*v-test* = *2.66*, *p* < *0.01*) **Model presentation **(**v-test = − 2.66**, ***p* < 0.01**)
3	8	*A-call *(*v-test* = *2.86*, *p* < *0.01*) **B-call **(**v-test = − 2.82**, ***p* = 0.02**)					*Yes *(*v-test* = *3.62*, *p* < *0.01*) **No **(**v-test = − 3.62**, ***p* < 0.01**)						
4	5	*B-call* (*v-test* = *2.94*, *p* < *0.01*) **A-call **(**v-test = − 2.43**, ***p* = 0.01**)	**v-test = − 2.20**, ***p* = 0.03**	*v-test* = *2.32*, *p* = *0.02*					*A-group* (*v-test* = *2.13*, *p* = *0.03*)				
5	11	*B-call *(*v-test* = *5.10*, *p* < *0.01*) **A-call **(**v-test = − 4.20**, ***p*** < **0.01**)	**v-test = − 3.63**, ***p* < 0.01**	*v-test* = *5.04*, *p* < *0.01*	*v-test* = *5.77*, *p* < *0.01*	*v-test* = *5.96*, *p* < *0.01*	*Yes *(*v-test* = *4.53*, *p* < *0.01*) **No **(**v-test = − 4.53**, ***p* < 0.01**)	*v-test* = *2.94*, *p* < *0.01*		*v-test* = *3.03*, *p* < *0.01*	*Felid *(*v-test* = *3.73*, *p* < *0.01*) **Raptor **(**v-test = − 3.73**, ***p* < 0.01**)		*Model presentation *(*v-test* = *3.88*, *p* < *0.01*) **Playback **(**v-test = − 3.88**, ***p* < 0.01**)

Values in italics are positively associated with the cluster, i.e. modalities of categorical variables are over-represented in the cluster, or the mean of numerical variables is greater than that of the population. Values in bold are negatively associated with the cluster. Only significant variables (*p* value < 0.05) are displayed for clarity

The sequence variables that were the most important to cluster vocal reactions were the type of first call emitted (chi-squared test, *df* = 8, *p* < 0.01) and the presence of loud calls (chi-squared test, *df* = 4, *p* < 0.01). Clusters were characterized by all the sequence variables: the first call emitted, the proportion of A- and B-calls in the first 20 s of emission, the number of calls emitted within 20 s, the duration of the vocal response, the emission of loud vocalizations and the proportion of individuals to respond (Table 1).

The contextual parameters that had the most important effect on the clustering of the vocal reactions were the predator type (chi-squared test, *df* = 4, *p* < 0.01) and the experimental paradigm (chi-squared test, *df* = 4, *p* < 0.01) (Fig. 2, Online Resource 2). The clusters could be characterized with four contextual parameters: the group identity, the number of individuals involved, the predator type and the experimental paradigm (Table 1).

## Discussion

We identified five main types of vocal reactions in response to predator experiments. The first vocal reaction type was characterized by the absence of a vocal response to playback stimuli. The second reaction type was characterized by sequences beginning with A-calls and mostly composed of A-calls, with a low emission rate, a short duration and absence of loud calls, mainly given to the raptor model and playbacks of both predator types. The third reaction type was characterized by sequences beginning with A-calls and comprising loud calls. This vocal reaction was not associated with a specific situation. The fourth reaction type was composed of sequences beginning with a B-call, with a high proportion of B-calls (and a low proportion of A-calls), mostly given by one group of monkeys. The fifth reaction type was characterized by sequences beginning with a B-call and mostly composed of B-calls, with a high rate of emission and presence of loud calls. These sequences lasted long and most individuals from the groups participated. They were only given to model presentations of felids.

Alarm vocal reaction of titi monkey groups was greatly influenced by the type of predator. This is congruent with recent studies suggesting that vocal reactions of individual titi monkeys to predator models were mostly influenced by predator type (Berthet et al. 2019b; Narbona Sabaté et al. 2022). Other studies (Cäsar et al. 2013; Berthet et al. 2019b) found that the vocal reaction of individual titi monkeys was affected by the location of the predator model, which was not our case. This could be explained by our restricted choice of descriptive sequence parameters, driven by the analysis at the group level and not the individual level. Some sequence parameters that encode for predator location, like the proportion of combinations of B-calls (Berthet et al. 2019b; Narbona Sabaté et al. 2022), could not be included here. Further studies need to isolate individual contributions and conduct finer sequential analyses using all relevant parameters.

The duration of the reaction was shorter for playbacks of predator calls than for presentations of visual models. This result is congruent with that of Arnold et al. (2008), who found that vocal responses to leopard playbacks were shorter than those to leopard models. This result is probably influenced by the experimental design itself. Indeed, subjects were exposed to the predator calls during the length of the playback stimulus (about 21 s), while they were exposed to the predator model for as long as they stayed in its vicinity (up to 2 h, Cäsar et al. 2013). This long exposure, combined with the fact that the predator model is not reacting to the mobbing of the monkeys, is likely responsible for the longer reaction to visual stimuli.

Interestingly, vocal reactions of titi monkeys were also significantly affected by the experimental paradigm. Specifically, titi monkeys were less likely to respond to playback experiments (cluster 1). They also displayed a specific vocal response to felid models (cluster 5), while vocal reactions to raptor models, raptor playbacks and felid playbacks were similar (cluster 2 and, to a lesser extent, clusters 3 and 4). In other words, titi monkeys exhibited a similar vocal reaction to raptor playback and raptor model presentations, but their reaction to playbacks of felids was different to that to felid models. This is congruent with the study of Adams and Kitchen (2020), which showed that behavioural reaction of saki monkeys varied with the exposure paradigm for jaguar, but not for harpy eagles. Several hypotheses can be put forward to explain our results.

First, since hunting felids do not vocalize, it is possible that titi monkeys exposed to felid calls infer that the predator is not hunting and does not represent an urgent threat, unlike the visual encounter with a silent cat (Ouattara et al. 2009a). Under this hypothesis, titi monkeys are either not signalling the presence of the predator (cluster 1) or do not signal that the predator is a felid (i.e. give a reaction different from cluster 5). This seems maladaptive: not reliably informing conspecifics about the presence of a predator, even if it is not in a hunting position, is highly risky, especially in a social system consisting of a family unit (i.e. a bonded, strictly monogamous couple and their offspring) (Dolotovskaya et al. 2020). It can be argued that the lack of vocal response (cluster 1) is a cryptic strategy, but the fact that, in some trials, groups responded with a vocal reaction also given to raptors (clusters 2, 3 and 4) does not support this idea. It can also be argued that titi monkeys do not react in a predator-specific way to felid playbacks because, since these predators can attack both from the ground and the canopy, acoustic cues leave the monkeys with little information about the location of the threat, and hence, the appropriate reaction to adopt is not straightforward (Adams and Kitchen 2020). This hypothesis does not apply here, for predator calls were broadcasted from distinct locations (ground or canopy) that left little room for life-threatening uncertainty about the predator’s location.

The second hypothesis is that playbacks of predator calls inform the whole group about the presence of a predator; hence, the subject may not need to further signal its presence. This hypothesis can explain why some groups remained silent upon hearing the felid playbacks (cluster 1). However, it does not explain why some other groups responded to felid playbacks with vocal reactions similar to those given to raptors (cluster 2). Moreover, if emitting alarm vocalizations after hearing a predator call was redundant, then titi monkeys should not emit vocalizations in response to raptor calls.

The third hypothesis is that titi monkeys do not recognize the vocalizations of felids. Less than half of studies conducting felid playbacks showed that the prey considered the vocalizations as threatening events (Hettena et al. 2014), which does not necessarily imply that they recognized the predator species. Moreover, several primate species do not seem to recognize their current predators’ calls (e.g. Hettena et al. 2014; Deppe 2020). Felid vocalization recognition is mostly learnt, but since cats are low-density, solitary animals that rarely vocalize, especially when hunting, learning opportunities are scarce (Hettena et al. 2014). This, combined with the fact that titi monkeys live in a dense habitat with low visibility, suggests that they can hardly attribute vocalizations of felids to their actual predators. On the contrary, raptors are encountered daily, mostly occur in open canopies, and often vocalize while hunting, making it easier to learn their calls (Barrera et al. 2011; MB, pers. obs.). The hypothesis that titi monkeys cannot recognize felid vocalizations is supported by the vocal reactions given to felid calls: while some groups did not respond at all to the playbacks, as if they did not recognize them as a threatening event (cluster 1), others responded as they do for raptors: they either considered it a novel sound, thus potentially threatening, or recognized that it was a threatening event but did not recognize that the predator was a felid (clusters 2, 3 and 4).

Further investigation is needed to refine the exact semantics of the titi monkeys’ vocal reactions, but these results provide interesting new leads. One hypothesis is that sequences given in response to raptor and felid calls (clusters 2, 3 and 4) convey information about a general, noteworthy event, as opposed to vocal reactions that convey specific information about the presence of a felid (cluster 5) (Dezecache and Berthet 2018). Another hypothesis is that alarm sequences convey information about the urgency of the threat. Raptors are thought to be the most dangerous threat to titi monkeys (Ferrari 2009). Reacting to unknown vocalizations (i.e. felid vocalizations) as if it was a highly dangerous threat (i.e. similar reaction to when encountering a raptor) can reflect a “better safe than sorry” strategy. If so, alarm sequences given to raptors and unknown stimuli may inform conspecifics about highly dangerous events (clusters 2, 3 and 4), and sequences given during visual encounters with felids (cluster 5) convey information about less dangerous events (Berthet et al. 2019a).

Playback presentations are often seen as an easy alternative to model presentation experiments, especially in wild settings. However, they do not always represent a perfect substitute, especially when investigating communication capacities of nonhuman animals. Our study complements others on American monkeys (Kemp and Kaplan 2011), African monkeys (Arnold et al. 2008; Ouattara et al. 2009a, b, c, d; Schel et al. 2009), lemurs (Fichtel 2007) and birds (Billings et al. 2015), which showed that prey sometimes emit different vocal reactions to visual and acoustic predator cues. We strongly encourage future work to systematically compare vocal reactions given to predator model presentations and to playbacks of predator vocalizations. Conclusions can be derived from the results using three predictions.

*If the paradigm does not influence the vocal reaction*, then it can be concluded that (i) both paradigms can safely be used interchangeably, and (ii) information encoded in the sequence is accessible via both visual and auditory modalities.

*If the reactions differ between paradigms, but most individuals emit similar vocal reactions*, then it can be concluded that (i) both paradigms cannot be used interchangeably, and (ii) the information encoded in the vocal utterances is retrieved differently depending on the sensory modality. For example, when exposed to a visual predator, the prey may easily collect important information, such as the location or distance of the predator, and reliably encode this in their alarm reactions. In playbacks, prey never access this knowledge, as they never find the predator. Moreover, the threat may be perceived as closer or more imminent—and thus more dangerous—when visual contact is established. Subjects are also in longer contact with the predator during model presentations, which can also influence information encoded in their vocal reactions. Finally, acoustic stimuli are heard by the entire group: all members simultaneously have access to the same information, which may alter the message to conspecifics, or even the necessity to inform others about the predator event (Ouattara et al. 2009a).

*If the reactions differ between paradigms and individuals do not emit similar vocal reactions* (which was our case here), it can be concluded that (i) both paradigms cannot be used interchangeably, and (ii) subjects fail to access relevant information about the event in one of the presentation modalities. For example, while prey are good at visually recognizing their predators, it is not sure that they always recognize their vocalizations (Blumstein 2000): this can represent a serious problem when one wants to investigate what information about the predatory event is encoded in the vocal response.

In sum, playbacks of predator vocalizations should not be used alone when investigating the communicative capacities of prey. Combining predator model presentations and playbacks of predator vocalizations, on the other hand, seems to be a powerful strategy to disentangle the cognitive mechanisms underlying communication in a large number of prey species. Controlling how the exposure paradigm influences the structure and information encoded in anti-predator vocal reactions can allow to perform fine-grained analyses on the semantic and syntactic capacities of nonhuman animals.

## Supplementary Information

Below is the link to the electronic supplementary material.

Supplementary file1 (DOCX 24 KB)

## Data Availability

The set of playback stimuli, the pictures of model stimuli, the set of raw recordings collected during the experiments, the complete dataset and the statistical R script are accessible on an online repository: https://figshare.com/projects/Impact_of_predator_model_presentation_paradigms_on_titi_monkey_alarm_sequences/138231.
