# Supplementary material for: Impact of predator model presentation paradigms on titi monkey alarm sequences
Source: Behav Ecol Sociobiol. 2022 Oct 3;76(11):143. doi: 10.1007/s00265-022-03250-1 (PMC9527189; doi:10.1007/s00265-022-03250-1)
Supplement: Supplementary file 1 — Supplementary file1 (DOCX 24 KB) [file 265_2022_3250_MOESM1_ESM.docx]

**Behavioral Ecology and Sociobiology**

**Supplementary material: Impact of predator model presentation paradigms on titi monkey alarm sequences**

Authors: Mélissa Berthet, Geoffrey Mesbahi, Cristiane Cäsar, Klaus Zuberbühler

Corresponding author: Mélissa Berthet, melissa.berthet.ac@gmail.com; Department of Comparative Language Science, University of Zurich, Switzerland; Center for the Interdisciplinary Study of Language Evolution (ISLE), University of Zurich, Switzerland

| Group | Number of individuals | Paired adults | Unpaired adults | Subadults | Juveniles | Infants |
| --- | --- | --- | --- | --- | --- | --- |
| A | 6 | 2 | 2-3 | 1 | 0-1 | 0-1 |
| D | 4-5 | 2 | 0-1 | 1 | 0-1 | 0-1 |
| R | 4-6 | 2 | 1-2 | 0-1 | 0 | 0-1 |
| M | 5-6 | 2 | 0-2 | 1 | 1 | 0-1 |
| P | 5-6 | 2 | 1-2 | 0-1 | 0-1 | 0-1 |
| S | 4-5 | 2 | 1-2 | 0-1 | 0 | 0-1 |

**Online Resource 1** Composition of the six groups of wild black-fronted titi monkeys *Callicebus nigrifrons* during the study period (2015-2016). We considered an individual as an adult from the age of 30 months, as a sub-adult between 18 and 30 months, as a juvenile between 6 and 18 months, and as an infant if less than 6 months old (Cäsar 2011).

| Experimental paradigm | Predator type | Predator location | Proportion of sequences | | Proportion of sequences whose first call was | | | Mean proportion of A-calls | Mean proportion of B-calls | Mean duration (s) | Mean number of calls emitted in 20 seconds | Mean number of individuals involved | Mean proportion of responding individuals | Number of sequences |
| --- | --- | --- | --- | --- | --- | --- | --- | --- | --- | --- | --- | --- | --- | --- |
|  |  |  | with loud calls | without loud calls | A | B | none |  |  |  |  |  |  |  |
| Playback | Raptor | Ground | 0.00 | 1.00 | 0.67 | 0.00 | 0.17 | 0.54 | 0.00 | 14.14 | 2.17 | 2.83 | 0.50 | 6 |
|  |  | Canopy | 0.00 | 1.00 | 1.00 | 0.00 | 0.00 | 0.91 | 0.00 | 16.43 | 4.17 | 2.50 | 0.71 | 6 |
|  | Felid | Ground | 0.17 | 0.83 | 0.50 | 0.17 | 0.33 | 0.38 | 0.26 | 72.19 | 4.50 | 3.17 | 0.46 | 6 |
|  |  | Canopy | 0.17 | 0.83 | 0.33 | 0.33 | 0.33 | 0.26 | 0.27 | 33.41 | 4.17 | 2.67 | 0.37 | 6 |
| Model presentation | Raptor | Ground | 0.50 | 0.50 | 0.67 | 0.33 | 0.00 | 0.65 | 0.35 | 451.42 | 6.00 | 3.17 | 0.87 | 6 |
|  |  | Canopy | 0.40 | 0.60 | 1.00 | 0.00 | 0.00 | 1.00 | 0.00 | 1010.01 | 6.40 | 3.60 | 0.68 | 5 |
|  | Felid | Ground | 1.00 | 0.00 | 0.17 | 0.83 | 0.00 | 0.01 | 0.99 | 3444.53 | 40.67 | 4.67 | 1.00 | 6 |
|  |  | Canopy | 1.00 | 0.00 | 0.00 | 1.00 | 0.00 | 0.05 | 0.94 | 4441.08 | 44.50 | 4.17 | 1.00 | 6 |

**Online Resource 2** Description of the titi monkey vocal reactions, averaged by experimental paradigm, predator type and location. If no monkey vocally reacted to the experiment, we coded the proportion of A- and B-calls in the 20 first seconds, the total duration, the number of calls emitted in the 20 first seconds, and the proportion of responding individuals as 0. Each of the six monkey groups provided one sequence per combination of paradigm and predator type and predator location, except for the presentation of raptor models in the canopy (the trial failed for the D group).

| Experimental paradigm | Predator type | Cluster 1 | Cluster 2 | Cluster 3 | Cluster 4 | Cluster 5 | Total |
| --- | --- | --- | --- | --- | --- | --- | --- |
| Playback | Raptor | 1 | 10 | 0 | 0 | 0 | 11 |
|  | Felid | 4 | 3 | 2 | 3 | 0 | 12 |
| Model presentation | Raptor | 0 | 4 | 5 | 2 | 0 | 11 |
|  | Felid | 0 | 0 | 1 | 0 | 11 | 12 |
| Total | | 5 | 17 | 8 | 5 | 11 | 46 |

**Online Resource 3** Clustering of the titi monkey vocal reactions, depending on the experimental paradigm and the predator type.
